# MicroRNA expression analysis identifies a subset of downregulated miRNAs in ALS motor neuron progenitors

**DOI:** 10.1038/s41598-018-28366-1

**Published:** 2018-07-04

**Authors:** Mafalda Rizzuti, Giuseppe Filosa, Valentina Melzi, Luca Calandriello, Laura Dioni, Valentina Bollati, Nereo Bresolin, Giacomo Pietro Comi, Silvia Barabino, Monica Nizzardo, Stefania Corti

**Affiliations:** 1 Dino Ferrari Centre, Neuroscience Section, Department of Pathophysiology and Transplantation (DEPT), University of Milan, Neurology Unit, IRCCS Foundation Ca’ Granda Ospedale Maggiore Policlinico, Milan, Italy; 2 Department of Biotechnology and Biosciences, University of Milano-Bicocca, Milan, Italy; 3 EPIGET LAB, Department of Clinical Sciences and Community Health, University of Milan, Unit of Occupational Medicine, IRCCS Foundation Ca’ Granda Ospedale Maggiore Policlinico, Milan, Italy

## Abstract

Amyotrophic lateral sclerosis (ALS) is a fatal neurological disorder that is characterized by a progressive degeneration of motor neurons (MNs). The pathomechanism underlying the disease is largely unknown, even though increasing evidence suggests that RNA metabolism, including microRNAs (miRNAs) may play an important role. In this study, human ALS induced pluripotent stem cells were differentiated into MN progenitors and their miRNA expression profiles were compared to those of healthy control cells. We identified 15 downregulated miRNAs in patients’ cells. Gene ontology and molecular pathway enrichment analysis indicated that the predicted target genes of the differentially expressed miRNAs were involved in neurodegeneration-related pathways. Among the 15 examined miRNAs, miR-34a and miR504 appeared particularly relevant due to their involvement in the p53 pathway, synaptic vesicle regulation and general involvement in neurodegenerative diseases. Taken together our results demonstrate that the neurodegenerative phenotype in ALS can be associated with a dysregulation of miRNAs involved in the control of disease-relevant genetic pathways, suggesting that targeting entire gene networks can be a potential strategy to treat complex diseases such as ALS.

## Introduction

Amyotrophic lateral sclerosis (ALS) is the most common and severe form of motor neuron disease (MND) in adults^1^. It is a fatal neurodegenerative disorder that affects motor neurons (MNs) leading to progressive muscle weakness and atrophy. Death usually occurs within 3–5 years after diagnosis due to respiratory failure^1,2^. Currently, due to the complexity of its etiopathogenesis and poor knowledge, there is no effective treatment and patients can rely only on supportive care and on Riluzole and Edaravone, the only two drugs approved for ALS treatment, which modestly prolong patient survival^3^.

The pathomechanisms underlying the disease are multifactorial and due to a complex interplay between genetics and environmental components, such as toxic exposure, diet and circulating inflammatory cytokines^4^. Patients without a familial history are generally recognized as sporadic (sALS) and account for the majority of cases, while familial forms of the disease (fALS) represent only 10% of clinical records^5^. To date, the most relevant genes associated with the disease are *C9ORF72, SOD1, TARDBP* and *FUS*, though several mutations in other genes have been reported to be involved in ALS pathogenesis^6,7^.

Currently, RNA pathway dysregulation appears to be a major contributor to ALS etiopathogenesis. Indeed, mutations in *C9ORF72*, which is the most common gene associated with ALS, lead to a toxic mRNA gain of function through RNA foci formation, and the subsequent sequestration and altered activity of RNA-binding proteins (RBPs)^8^. TDP-43 and FUS are also deeply involved in RNA metabolism. In a pathological context, such as cellular stress, the association between TDP-43, FUS and the mRNA can lead to aberrant phosphorylation, ubiquitination and the aggregation of proteins, as well as formation of stress granules (SGs)^9^. Aggregated RBPs are sequestered in SGs, leading to the formation of cytoplasmic inclusions and to the disruption of RNA processing^10^. Moreover, both TDP-43 and FUS have been implicated in microRNA (miRNA) processing^11^.

miRNAs are short evolutionarily conserved noncoding single-stranded RNA molecules that regulate gene expression via RNA-dependent post-transcriptional silencing mechanisms^12^. They target the mRNA 3′-UTR, causing the mRNA downregulation either through its destabilization or protein translation inhibition^13^. Indeed, the degree of miRNA-mRNA target complementarity determines the fate of the target mRNA: a perfect annealing leads to transcript degradation, while an incomplete base-pairing is associated with translational repression, mRNA degradation or sequestration into cytoplasmic structures named P-bodies^2^.

Since a single miRNA can target multiple genes and a group of miRNAs may regulate the same target gene, these short sequences appear to be involved in almost all biological processes^14–16^. Indeed, miRNAs are crucial in determining cell homeostasis and biological fate, and they are usually subject to modification during disease pathogenesis^17^. Particularly, the control of gene expression by miRNAs is important for the maintenance of neuron survival and physiological functions, so that dysfunctions in miRNA biogenesis can have severe consequences in neurological disease^18–20^.

miRNA biogenesis is a stepwise process strictly regulated by specific enzymatic complexes^7^. A surprising number of proteins associated with ALS are involved in miRNA processing^21,22^. For example, TDP-43 and FUS promote miRNA biogenesis by interacting with Drosha and Dicer, two key enzymes for processing miRNAs from precursors into mature molecules^23,24^. Moreover, mutations in *TDP-43*, *FUS*, and *SOD1* activate a stress response pathway that leads to general decreased miRNA levels, which most likely contributes to MN degeneration^25^. Indeed, several studies describe miRNA dysregulation in ALS pathology^7^.

Due to the important roles of miRNAs in the fine-tuning of crucial cellular functions, they could represent an important tool for promoting modulations in biological pathways, which could explain, at least partially, complex diseases such as ALS. In fact, addressing the biological consequences of aberrant miRNA levels could contribute to the elucidation of the molecular mechanisms that lead to MN degeneration, thereby expanding our understanding of ALS pathogenesis.

In this study, we used induced pluripotent stem cells (iPSCs) to investigate miRNA-mediated pathogenic mechanisms in ALS. Patient-specific stem cells represent a promising *in vitro* disease model for ALS research, since they can be differentiated to different cell types harbouring the same patients’ genomic backgrounds. We studied miRNA dysregulation in MN progenitors differentiated from fALS and sALS patient iPSCs and we identified a subset of 15 differentially expressed miRNAs in patient-derived cells compared to healthy ones.

Gene Ontology enrichment and Reactome pathway analyses highlighted that the most involved deregulated pathways are associated with disease-relevant mechanisms, including synaptic vesicle synthesis, release, reuptake and degradation, apoptosis and epigenetic regulation of gene expression. Among the identified miRNAs, miR-34a and miR504 were further analyzed due to their implication in cell cycle regulation via the p53 pathway and their already described downregulation in neurological disorders^26–30^. Overall, our results confirmed the crucial role for miRNA deregulation in neurodegeneration and ALS. The identification of common downstream genetic pathways controlled by candidate miRNAs can lead to the discovery of pathological mechanisms and the development of therapeutic strategies that target multiple gene networks, increasing the chances of modifying a multifactorial disease such as ALS.

## Results

### Generation of iPSCs lines and MN progenitors as an *in vitro* model of ALS

We reprogrammed fibroblasts from two sporadic ALS patients (sALS n = 2), two familial ALS cases with SOD1 mutations (fALS n = 2) and two healthy subjects as controls (CTRL n = 2) into iPSCs (Supplementary Table S1) using a non-integrating reprogramming protocol based on Sendai virus technology^31,32^. The obtained iPSCs expressed typical stem cell markers including OCT4, SOX2, SSEA4 (Supplementary Fig. S1).

**Figure 1:**
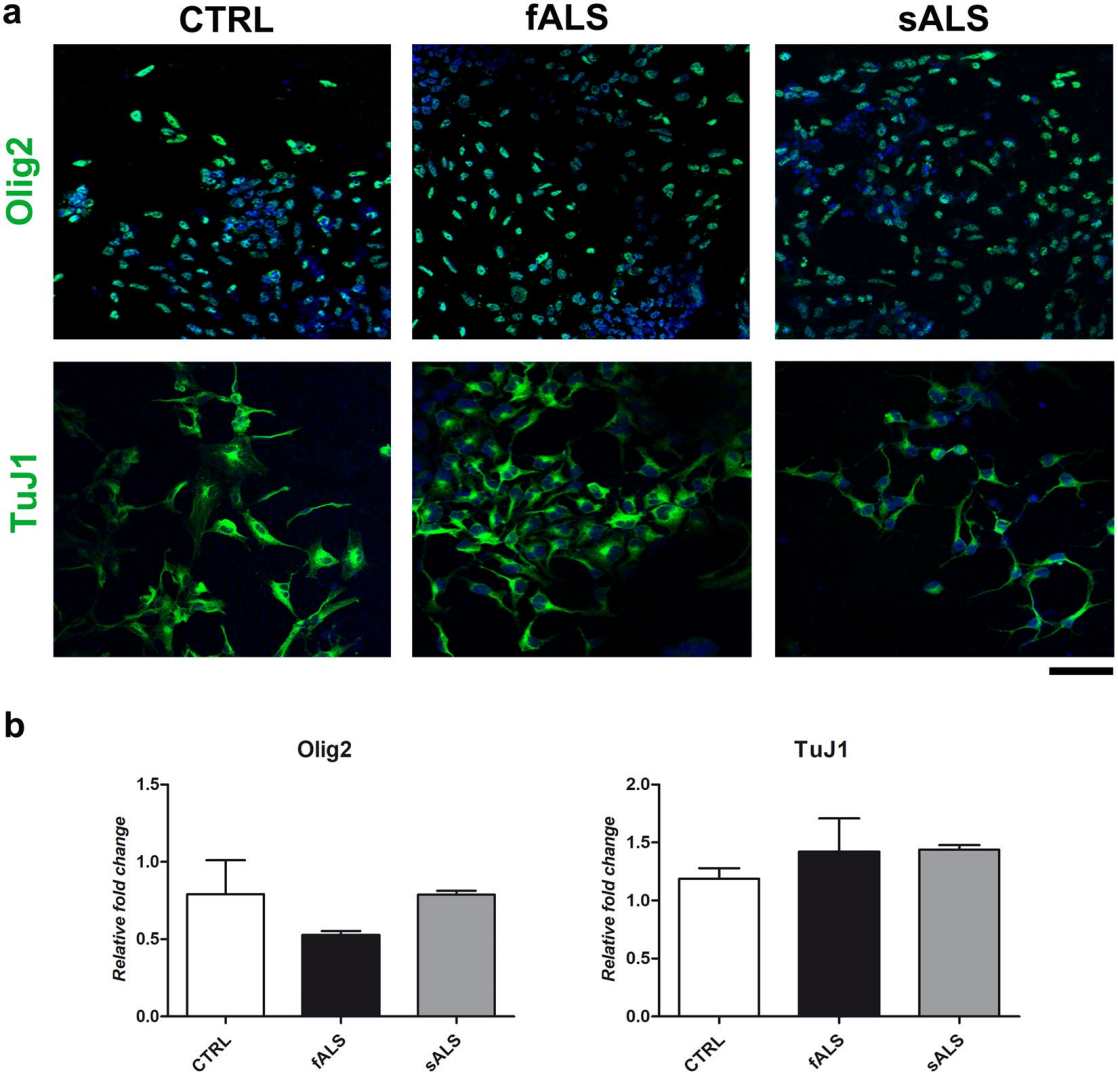
Immunocytochemistry (ICC) and qPCR performed on MN progenitors. (**a**) MN progenitors differentiated from fALS, sALS and control iPSCs (CTRL) expressed typical neuronal markers (Olig2, green; TuJ1, green). Nuclei are counterstained with DAPI (blue signal). Scale bars: Olig270 µm and TuJ1 50 µm. (**b**) Quantification of *Olig2* and *TuJ1* by qPCR performed on fALS, sALS and control (CTRL) MN progenitors (student t-test, values represent means + SEM).

We differentiated ALS and CTRL-iPSCs using a specific dual SMAD inhibition protocol based on neural induction and exposure to a combination of small molecules that leads to the formation >90% MN progenitors in a 14-day time frame^33^. These cells were positive for lineage-specific markers including Olig2 and βIII Tubulin (TuJ1) (Fig. 1a). Quantitative PCR showed that expression levels of Olig2 and TuJ1 were comparable among control, sALS, and fALS cell lines, suggesting there is no difference in the proportion of MN progenitors among these lines (Fig. 1b).

### miRNA expression profiles of MN progenitors show a miRNAs downregulation in ALS cells compared to controls

We next profiled the miRNA transcriptome of MN progenitors using the TaqMan® Low Density Array, which enabled the accurate quantitation of 754 human miRNAs.

**Figure 2:**
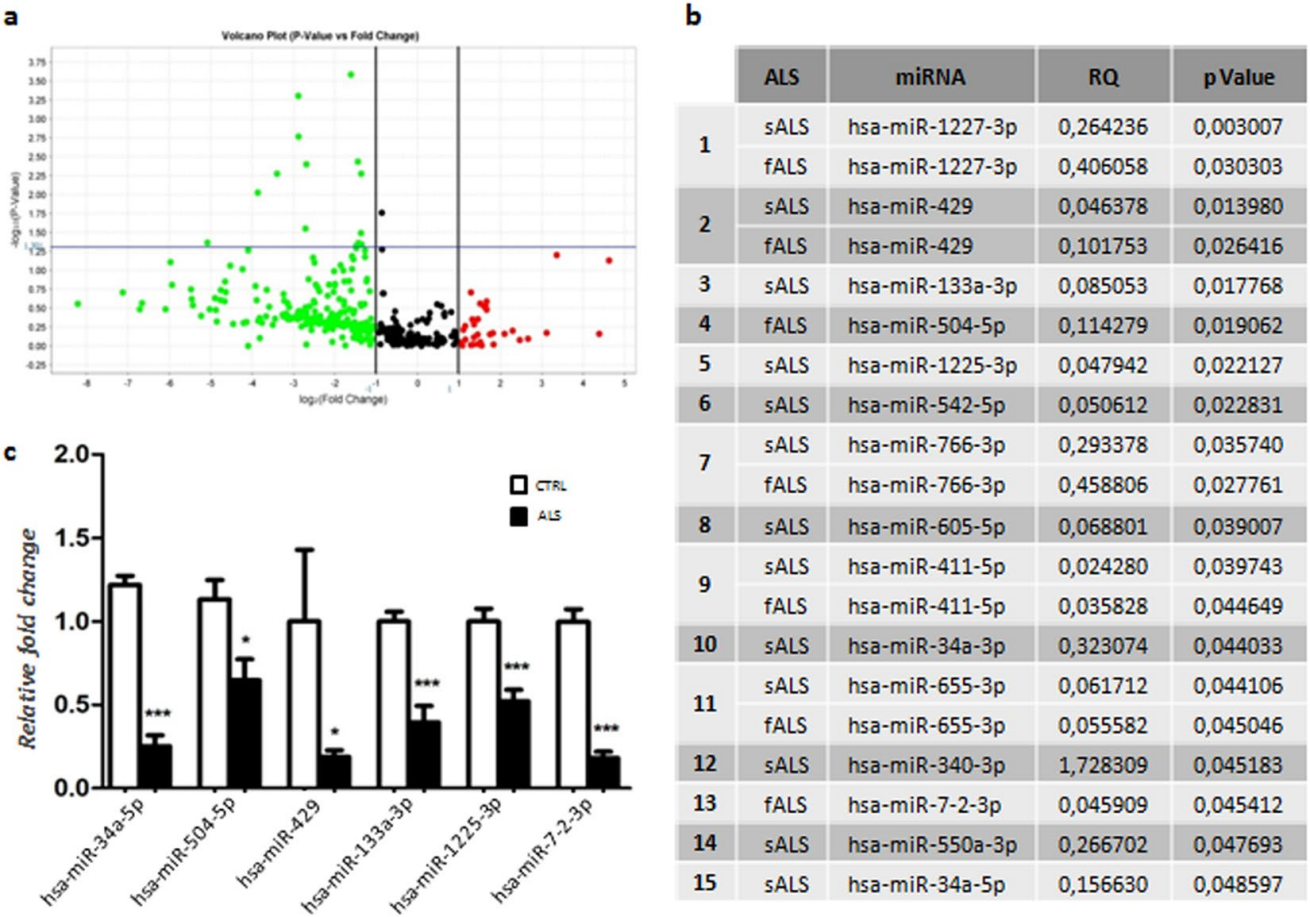
MiRNA expression profile in ALS patients versus healthy controls. (**a**) Volcano plot data from TaqMan® Assays on microfluidics cards; data presented include sALS and fALS subjects in a unique biological group (ALS). (**b**) List of downregulated miRNAs in ALS-MN progenitors from the volcano plot analysis. (**c**) Validation of data derived from the microfluidics cards with specific qPCR assays confirmed the downregulation of miR-34a, miR-504, miR-429, miR-133a, miR-1225-3p, and miR-7-2* in ALS-MN progenitors (ALS) (***P < 0.0001 and *P < 0.05, student t-test, values represent means + SEM) compared to the controls (CTRL).

The analysis was performed on ALS-MNs progenitors (n = 4, enclosing sALS and fALS samples in a unique biological group) versus CTRL-MNs (n = 2). We observed a decreased expression of 15 miRNAs in ALS-MN progenitors (Fig. 2a,b). We confirmed the results obtained with the Human Array MicroRNA cards using specific qPCR assays performed on a random subset of identified miRNAs (Fig. 2c, Supplementary Fig. S2). In particular, we tested miR-504 (*P* < 0.05), miR-429 (*P* < 0.05), miR-34a (*P* < 0.0001), miR-133a (*P* < 0.0001), miR-7-2* (*P* < 0.0001) and miR-1225-3p (*P* < 0.0001) (Fig. 2c).

### Bioinformatics analysis on dysregulated miRNAs identifies deregulated pathways in ALS-MN progenitors

To identify target genes associated with the identified miRNAs we interrogated miRTarBase, one of the most comprehensively annotated and experimentally validated miRNA-target interactions databases, and Mirgate, which contains novel computationally predicted miRNA–mRNA interactions. We identified 2923 validated target genes in MiRTarBase and 1018 putative targets in Mirgate.

Then, to obtain a more reliable target dataset, we intersected the lists generated by both databases. Of the common 368 genes, 278 genes were targeted by the same miRNAs thus representing the most stringent and reliable dataset.

**Figure 3:**
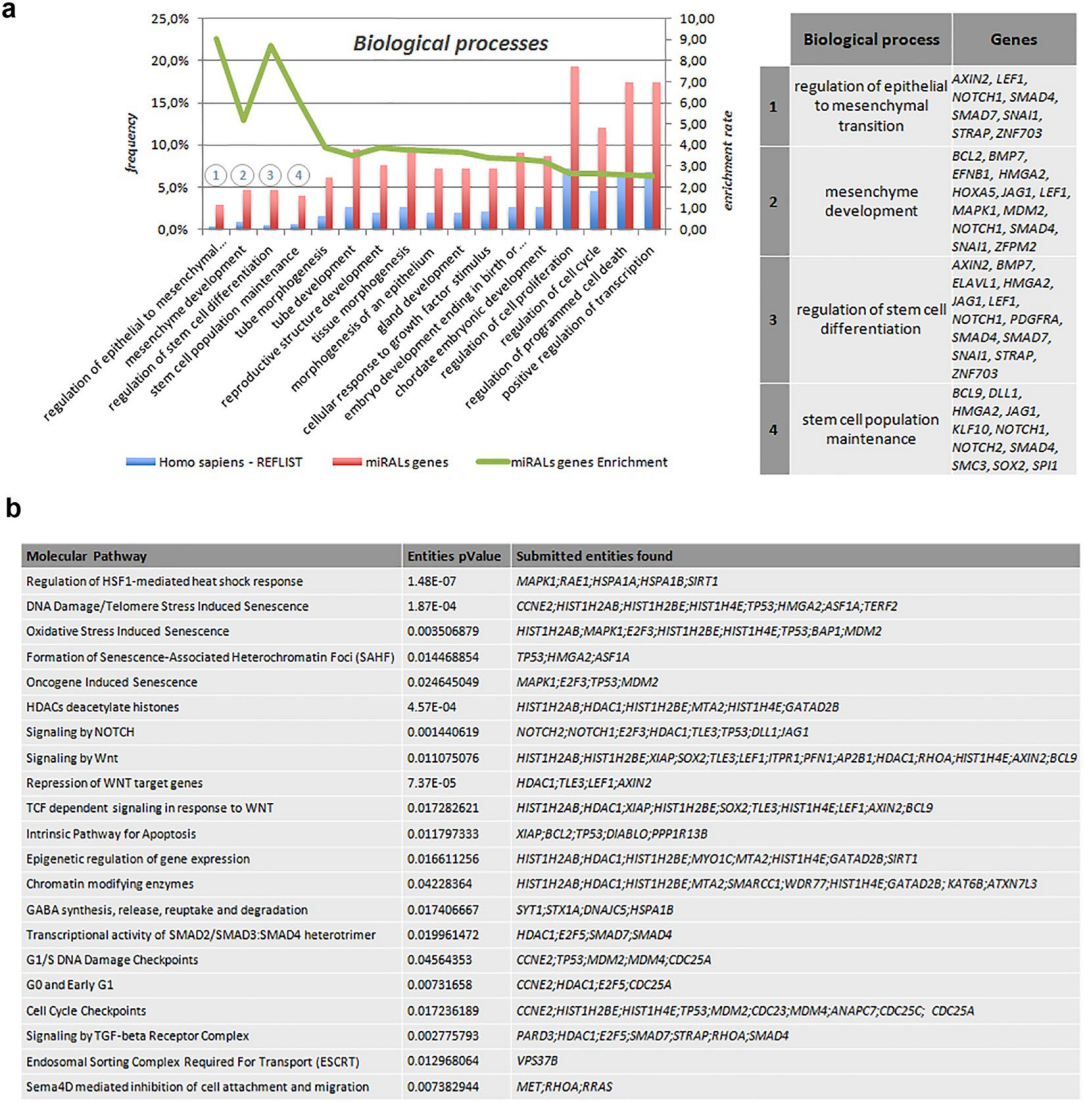
GO and Molecular Pathway analysis performed on the 278 genes identified by bioinformatics. (**a**) GO enrichment analysis showed the most significant biological processes enriched in ALS-MN progenitors (*P* < 0.05) and the list of genes associated with the 4 most enriched processes. **(b)** Table of selected pathways with the related genes emerging from the Reactome analysis (*P* < 0.05).

In order to comprehensively describe the properties of these 278 target genes, we first performed Gene Ontology Enrichment analysis (GO). The analysis of biological processes revealed that the selected miRNAs were mainly involved in stem cell-related processes, such as regulation of epithelial to mesenchymal transition, mesenchyme development, regulation of stem cells differentiation and stem cell population (Fig. 3a).

Next, we analyzed our group of candidate genes with Reactome pathway over-representation to investigate the associated molecular pathways. Interestingly, the analysis showed that the putative targets of the selected miRNAs were associated with several pathways including chromatin modifying enzymes; oxidative stress; induced senescence; GABA synthesis, release, reuptake and degradation (Fig. 3b).

Overall, these results suggest a potential role for miRNA reduction and particularly among those involved in neuronal processes and cell response injuries in ALS.

### Gene expression analysis showed a deregulation of selected target genes

**Figure 4:**
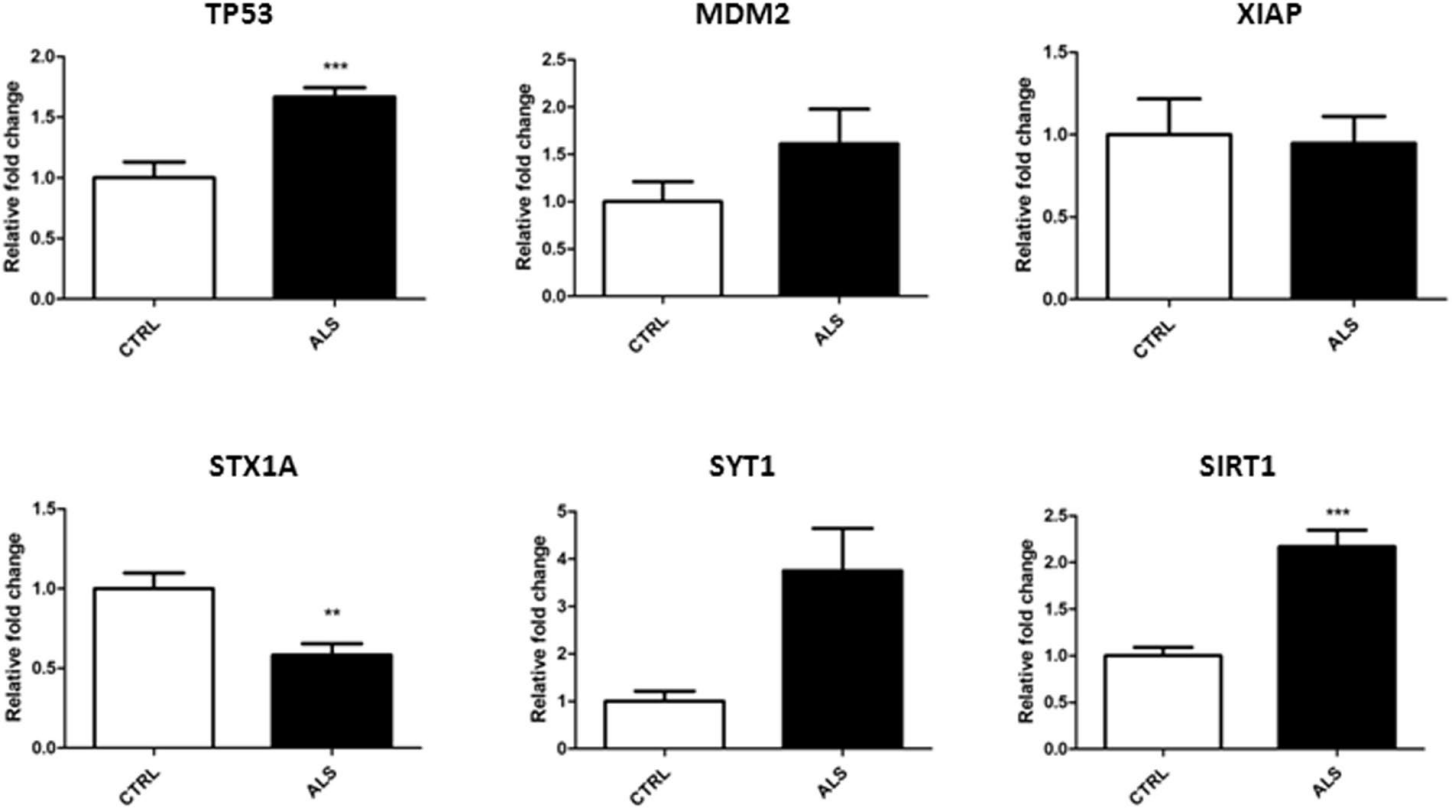
Gene expression analysis of selected target genes identified. Specific qPCR gene expression analysis for *SIRT1*, *STX1A*, *SYT1*, *XIAP*, *MDM2* and *TP53* (***P < 0.001 and **P < 0.01, student t-test, values represent means + SEM) in ALS cells (ALS) compared to the control cells (CTRL).

To experimentally characterize the involvement of the molecular pathways that had emerged from the Reactome analysis (Fig. 3b), we selected specific genes based on their expression in the central nervous system and their likely involvement in disease pathogenesis. We thus assayed, for quantitative Real Time PCR analysis (qRT-PCR) in ALS and CTRL-MN progenitors, *Tumor protein p53* (*TP53*), *MDM2 proto-oncogene* (*MDM2*), and *X-linked inhibitor of apoptosis* (*XIAP*), associated with oxidative stress-induced senescence and apoptosis; *Syntaxin 1A* (*STX1A*) and *Synaptotagmin 1* (*SYT1*), which are involved in synaptic vesicle trafficking; *Sirtuin 1 (SIRT1)*, a specific is target of miR-34a^28^ (Supplementary Table S2). As shown in Fig. 4, qRT-PCR analysis revealed increased expression of *TP53* (*P* < 0.001) and *MDM2*, while the transcript levels of *XIAP* did not change in the pathological samples. We further detected a significant reduction in *STX1A* transcript levels (*P* < 0.01), while we observed an increase in *SYT1* albeit not statistically significant. Notably, all these genes are regulated by miR-34a and/or miR-504 suggesting that these miRNAs may have a key role in ALS pathology. Finally, consistent with the downregulation of miR-34a that we observed in our ALS-MN progenitors, we identified a significant upregulation of *SIRT1* (*P* < 0.001).

Overall, these results further support the potential role of these genes as downstream targets of miR-34a and miR-504 in ALS pathogenesis.

### Enoxacin treatment rescues miR-34a levels in ALS-iPSCs

**Figure 5:**
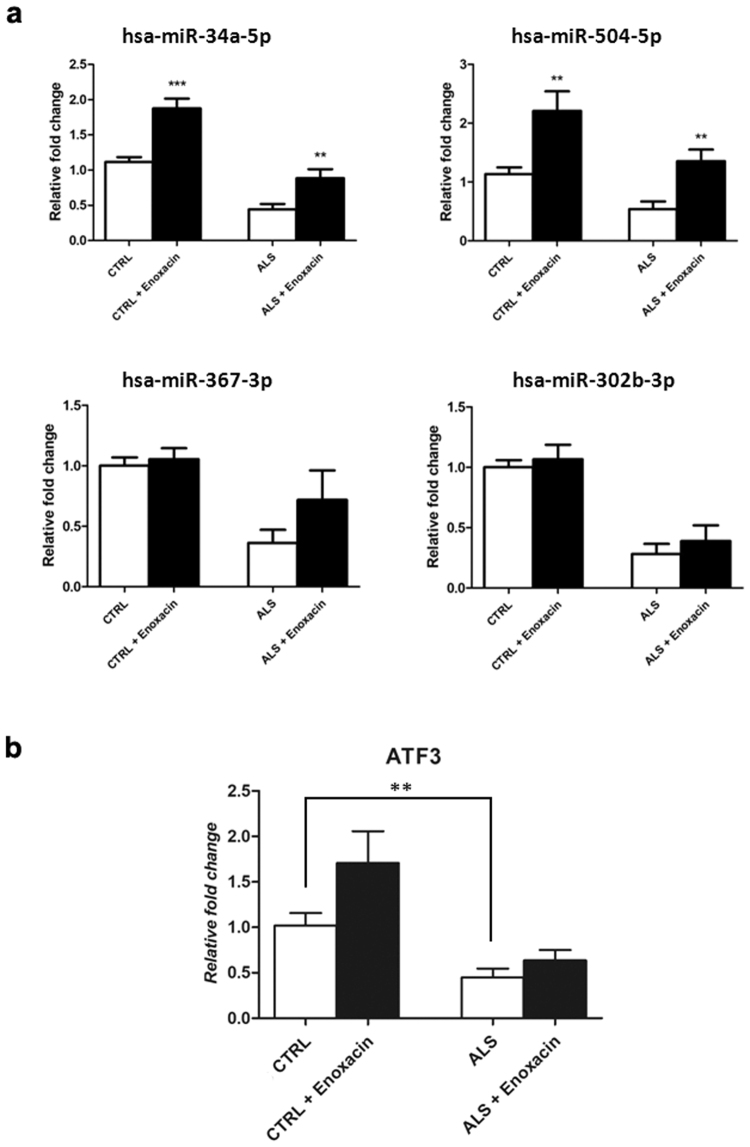
miRNA expression analysis in control and ALS iPSCs and *ATF3* quantification after enoxacin treatment. (**a**) qPCR experiments performed on miR-34a, miR-504, miR-367 and miR-302b in control (CTRL) and ALS iPSCs (ALS) treated or not treated with 100 μM enoxacin for 48 hours. Enoxacin treatment increased the amount of miRNAs in both control and ALS iPSCs (***P < 0.0001 and **P < 0.01, student t-test, values represent means + SEM). The experiment was repeated three times. (**b**) qPCR experiments performed on *ATF3* in control (CTRL) and ALS-MN progenitors (ALS) treated or not treated with 100 μM enoxacin for 48 hours. ALS displayed significant low levels of *ATF3* (**P < 0.01, student t-test, values represent means + SEM).

According to previous reports^7,25^ we observed a preferential downregulation of miRNAs in ALS patients-derived cell lines. Because this reduction of miRNA levels may contribute to MN degeneration^18,25^, we examined the effect of enoxacin, a drug that was previously shown to increase miRNA levels^25,34^. Enoxacin is a fluoroquinolone antibiotic able to enhance the binding affinity of TRBP to pre-miRNAs, thereby increasing the activity of the pre-miRNA processing factor Dicer^25,35,36^. Importantly, enoxacin received an orphan designation for ALS treatment by the European Medicine Agency (EU/3/15/1459) because of its ability to improve miRNA biogenesis^36^. Among the identified miRNAs, we specifically focused on miR-34a and miR-504 due to their involvement in neurodegenerative pathways. As an internal control, we used miR-302 and miR-367, which are described in the literature as expressed in iPSCs under basal conditions. We analyzed the expression of the miRNAs in ALS-iPSCs versus control iPSCs either in the absence or in the presence of enoxacin. Consistent with the described effect of enoxacin, we detected an increased expression of all tested miRNAs, which was statistically significant for miR-34a and miR-504 (*P* < 0.01, Fig. 5a). Finally, we investigated whether enoxacin treatment could affect the levels of some of the proteins involved in miRNA biogenesis, such as Drosha, Dicer and Ago2. In general, we found that this drug did not appear to exert any effects on their protein levels (data not shown), confirming, as has already been reported in literature^25^, that enoxacin acts on Dicer activity and not on its protein level. We also demonstrated in our ALS-MN progenitors a downregulation of *activating transcription factor 3* (*ATF3*, *P* < 0.01), already demonstrated as implicated in ALS pathology^37^. This decrease resulted mitigated after enoxacin treatment (Fig. 5b). The same trend was observed by western blot analysis (data not shown).

## Discussion

The pathomechanisms underlying ALS are almost unknown, even though the role of alterations in RNA metabolism, including miRNA processing, has been increasingly recognized. miRNAs are tissue-specific small RNA molecules that can individually regulate several hundred targets. Since miRNAs are linked to gene regulatory networks implicated in neural induction, neuronal differentiation, and fate specification^38^, they can likely be implicated in MN development and in the etiology or progression of neurodegenerative diseases. Therefore, due to their role in controlling fundamental physiological functions in neurons^2,39^, the identification of deregulated miRNAs in pathological conditions could represent a strategy to understand disease mechanisms as well as to select novel therapeutic targets.

Growing evidence, such as recent data obtained for spinocerebellar ataxia type 1, suggests that adult-onset neurodegenerative diseases, including ALS, may also be rooted in development^40^. iPSC-based models allow the aetiopathology of ALS to be studied, including in the developmental stages that are otherwise inaccessible. For this reason, in this study, we derived MN progenitors^33^ directly from patient fibroblasts exploiting the iPSCs technology. Moreover, MN progenitors allowed us to characterize some molecular events related to ALS during the early stages of differentiation that can be important for the subsequent manifestation of ALS.

We performed miRNA profiling on MN progenitors through micro fluidic cards technology. Consistent with previous reports that observed a general downregulation of miRNAs in ALS^7^, we identified 15 significantly downregulated miRNAs in our ALS lines enclosing sALS and fALS samples as a unique biological group. Indeed, even if fALS and sALS can have different pathological mechanisms, we think that finding a common target deregulated in both sporadic and familiar patients can be useful either to understand the pathogenesis or to find a common therapeutic strategy. Bioinformatics analysis allowed the identification of 278 genes that are targeted by a common set of dysregulated miRNAs. Gene Ontology and Reactome pathway analysis showed that the main significantly enriched biological processes were specifically related to several pathways including programmed cell death, oxidative stress-induced senescence, synaptic vesicles synthesis, release, reuptake and degradation, apoptosis and epigenetic regulation of gene expression. Among the 15 miRNAs identified in our study, miR-34a and miR-504 were relevant candidates to be further investigated for target validation. We focused on genes that are involved in the main pathways that are consistent with degeneration processes occurring in ALS, namely in apoptosis, epigenetic regulation, and in synaptic vescicles metabolism.

Based on the Reactome analysis, miR-34a was associated with almost all the affected pathways and related genes. Literature evidence shows that this miRNA is involved in cell cycle regulation, induction of apoptosis after cell damage, and autophagy^29,41,42^. Intriguingly, miR-34a and miR-504 share many molecular pathways related to apoptosis^43,44^. miR-34a has already been found to be downregulated in the spinal cord and brain stem of SOD1G93A transgenic mice, at different stages of the disease and has been considered an apoptotic-related miRNA^30^, while miR-504 deregulation has not yet been described in the context of ALS. miR-34a is a direct transcriptional target of *TP53*^45^ gene while miR-504 is a direct upstream negative regulator of TP53 protein expression^43,44^. Moreover, the activation of *TP53* can result in transcription of miR-34, which in turn promotes *TP53* expression^44^. Finally, the promoter region of the miR-34 gene is regulated by epigenetic mechanisms and promoter demethylation can induce miR-34a expression, *TP53* transcription and the subsequent regulation of genes involved in cell cycle control^44^. Interestingly, TP53 and related proteins have already been associated with neurodegenerative diseases and motor neuronal cell death both in patients and in ALS murine models^46,47^. For these reasons, we further investigated the mRNA level of *TP53* and of *MDM2* and *XIAP*, two other apoptosis-related genes that were identified as miR-34a and miR-504 targets in our bioinformatics analysis. We found increased *TP53* and *MDM2* transcript levels in ALS-MN progenitors. MDM2 binds p53 and controls its transcriptional activity and stability through an autoregolatory feedback mechanism^48^. However, the simultaneous upregulation of *TP53* and *MDM2* has been already described in mouse skeletal muscles in ALS mice^49^. With regards to *XIAP*, we did not observe any significant differences in terms of gene expression in ALS cells compared to controls. It has been reported that *XIAP* is decreased in the disease and its overexpression in spinal cord neurons in ALS mice displayed beneficial effects on survival^50^.

Epigenetic processes can alter the genetic information as a result of environmental signals, and these mechanisms are particularly important in the neurodegenerative field, which makes the epigenome an attractive therapeutic target^51^. In ALS, it has been reported that several environmental insults can trigger the release of free radicals leading to oxidative stress, epigenetic modifications and finally changes in gene expression^52^. In this context, it is worth mentioning that the promoter region of the miR-34 gene is regulated by epigenetic mechanisms and promoter demethylation can induce miR-34a expression, TP53 activation and the regulation of various genes involved in the cell cycle^44^.

In yeast sirtuin proteins are known to regulate epigenetic gene silencing. The human *SIRT1* gene is a direct target of miR-34a^53^. We assayed the level of *SIRT1*, and observed a statistically significant upregulation consistent with the downregulation of miR-34a in ALS-MN progenitors. Interestingly, SIRT1 was shown to protect cells from oxidative stress^54^ and apoptosis^55^. Thus, increased expression of SIRT1 may represent a pro-survival response for ALS MN progenitors.

Based on the known role of synaptic vesicles at neuromuscular junctions, we investigated the expression of *STX1A* and *SYT1*, two genes identified in the Reactome analysis, targeted by miR-34, and involved in synaptic functions. In fact, synapses are basic structural and functional units of the central nervous system-muscles connection that are highly vulnerable to pathological conditions, particularly in neurodegenerative diseases. In ALS, mutations of *SOD1*, *TDP43* and *FUS* have already been linked to synaptic dysfunctions^56^. Particularly, presynaptic alterations appear to be early symptoms of neuronal disorders. Interestingly, our gene expression analysis confirmed the dysregulated expression of *STX1A*, a member of the syntaxin superfamily that is involved in neurotransmitter release at the presynaptic membrane. We found a significant decrease of *STX1A* levels in our ALS cells, which confirmed data already published about syntaxin family members in the spinal cord from ALS patients^57^. We also assayed *SYT1* levels since this protein acts as a key regulator for synaptic vesicle exocytosis and endocytosis, regulating their docking in response to the presence of calcium^58^ and because it has already been associated with synaptic transmission defects in another murine model of a MN disease, Spinal Muscular Atrophy (SMA)^59^. Moreover, *SYT1* downregulation has been reported in nerve terminals from highly affected muscles, which seems to be associated with SMA mouse muscle vulnerability^60^. However, in the spinal cords of SOD1 mice, higher SYT1 expression was observed and was linked to the altered calcium concentrations and ALS-linked excitotoxicity^61^. Of note, we confirmed an upregulation of *SYT1* transcript levels in our ALS-MN progenitors. Thus, our gene expression results for *STX1A* and *SYT1* support a key role for these proteins in synaptic vesicle trafficking changes in ALS that requires further investigations.

Finally, we investigated the potential therapeutic effect of enoxacin on miRNA levels in ALS. Enoxacin is an antibiotic that recently received an orphan designation for ALS treatment by the EMA (EU/3/15/1459) thanks to its ability to increase Dicer activity. Indeed, this compound enhances the binding affinity of TAR RNA binding protein to pre-miRNAs, thereby increasing pre-miRNA processing by Dicer^35,36^. Since Dicer is known to be crucial in miRNA biogenesis, enoxacin is expected to revert the general miRNA downregulation related to ALS disease. Therefore, we treated ALS and control iPSCs with enoxacin observing a slight increase in miRNAs levels in the treated cells compared to untreated cells. We have already shown that fully differentiated MNs in ALS had decreased survival that correlated with variations in the activation of the apoptotic pathway after long term culture^62^. Even if in the present study, we did not observe a difference among control, sALS, and fALS MN progenitors obtainment, we found decreased expression of *activating transcription factor 3* (*ATF3*). Expression of *ATF3* prevents cell death and promotes neurite formation and elongation that induces the expression of survival and growth-associated genes. Moreover, it has already been demonstrated that overexpression of *ATF3* in the MN of SOD1G93A mice supports their survival and axonal integrity maintenance^37^. Here, we demonstrated that enoxacin treatment may slightly enhance *ATF3* levels in ALS, suggesting that the general upregulation of miRNA levels can be beneficial by regulating key genes that modulate pathological phenotypes. Overall, our results highlight the crucial role of miRNAs in ALS physiopathology, providing insights into pathogenic mechanisms. In particular, we identified two miRNAs, miR-34a and miR504, as well as their downstream pathways, in particular apoptosis and synaptic vesicle regulation, that can account for ALS pathological mechanisms and represent potential therapeutic targets. The identification of common pathways associated with different deregulated miRNAs in ALS could be crucial for identifying therapeutic target. ALS is a complex disease that involves several biological pathways, and a suitable innovative and effective therapy likely has to rely on a multi-faceted approach.

These analyses should be further examined in additional cell lines obtained from patients carrying different mutations to detect shared pathogenic mechanisms. Moreover, miRNA-based therapies should be tested to confirm the relevance of the identified pathways.

## Material and Methods

### iPSCs generation

The studies involving human samples were conducted in accordance with the Code of Ethics of the World Medical Association (Declaration of Helsinki) and with national legislation and institutional guidelines. After obtaining informed consent, we reprogrammed fibroblasts derived from skin biopsies (Eurobiobank, ethical committee approval at the IRCCS Foundation Ca’ Granda Ospedale Maggiore Policlinico) from ALS (n = 2 sALS and n = 2 fALS subjects harbouring mutations in *SOD1*) and healthy subjects (n = 2) into iPSCs using the CytoTune®-iPS 2.0 Sendai Reprogramming Kit (Life Technologies, Carlsbad, CA, USA) according to manufacturer’s instructions. The kit contains 3 vectors derived from a modified and non-integrating form of Sendai virus (SeV) which carries the 4 Yamanaka’s factors Oct4, Sox2, Klf4 and c-Myc^63^.

Fibroblasts derived from subject’ biopsies were cultured in DMEM high glucose (Life Technologies, Carlsbad, CA, USA), with 15% fetal bovine serum (FBS) (Euroclone, Milan, Italy) and infected with Sendai viruses. After two weeks the first iPSC colonies were obtained, mechanically picked and maintained in Essential 8 Medium (Life Technologies, Carlsbad, CA, USA) on plates covered with a thin cultrex layer (Cultrex^®^ Stem Cell Qualified Reduced Growth Factor Basement Membrane Extract PathClear^®^; Thema Ricerca, Castenaso, Italy). Information about the patients from whom the iPSC lines were derived is provided in Supplementary Table S1.

We performed Enoxacin treatment in iPSC lines by adding 100 µM Enoxacin to cell media (RNAi Enhancer Enoxacin Sodium Salt; Millipore, Burlington, MA, USA). We collected samples 48 hours after treatment and we stored cell pellets at −80 °C.

### iPSC differentiation into MN progenitors

iPSCs were differentiated into MN progenitors using a 14 days multistep protocol. We slightly modified a previously published protocol^33^. Specifically, iPSCs were plated in neural induction medium (DMEM/F12, 1x Glutamax, 0.5x MEAA, and 1x penicillin/streptomycin) (Life Technologies, Carlsbad, CA, USA) supplemented with 10 µM SB (Sigma Aldrich, Saint Louis, MO, USA) and 0,2 µM LDN (Stemgent, Cambridge, MA, USA) for 7 days. We subsequently promoted MN progenitors’ formation by using a combination of small molecules that address specific caudal differentiation: 1 µM Retinoic Acid (RA; Sigma Aldrich, Saint Louis, MO, USA) and 200 ng/mL of Sonic Hedgehog (Shh; Sigma Aldrich, Saint Louis, MO, USA) were added for additional 7 days. Cells were collected and stored at −80 °C for the analysis.

### Immunocytochemistry analysis

Cells were fixed with 4% paraformaldehyde for 10 minutes, permeabilized with 0,25% TritonX-100 and subsequently blocked with 10% BSA and 0,3% Triton X-100 in 1x PBS solution, for 1 h at room temperature (RT). Slides were incubated overnight at 4 °C with specific primary antibodies and incubated with the appropriate secondary antibodies conjugated with Alexa-Fluor 488 or 568 (anti-mouse, rabbit or goat, 1:1000 Life Technologies, Carlsbad, CA, USA) for 1 hour and 30 minutes at RT. Image acquisition was performed with a LEICA LCS2 microscope.

The following antibodies and dilutions were used for iPSC staining: SSEA-4 (mouse 1:500, Chemicon International, Temecula, CA, USA), OCT4 (mouse 1:500, Chemicon, International, Temecula, CA, USA), and SOX2 (mouse 1:500, Chemicon, International, Temecula, CA, USA). For MN progenitor staining, we employed TuJ1 (anti-rabbit 1:400, Millipore, Burlington, MA, USA) and Olig2 (anti-mouse 1:100, Sigma Aldrich, Saint Louis, MO, USA) antibodies.

### Total RNA and miRNA isolation

For each sample, both total RNA and miRNA were extracted using *mir*Vana™ miRNA Isolation Kit (Life Technologies, Carlsbad, CA, USA). Total RNA was eluted in 70 µL of pre-heated (95 °C) Elution Solution. To separate large RNA species from the enriched small RNAs, we sequentially immobilized the large RNAs on two filter cartridges and finally collected the flow-through containing mostly the small RNA fraction. The purified small RNA fraction was eluted in 30 µL of pre-heated Elution Solution. To identify the RNA profile and obtain information about RNA integrity the miRNA samples were analyzed by 2100 Bioanalyzer (Agilent Tecnologies, Santa Clara, CA), using Agilent RNA 6000 Nano Kit. After miRNA isolation samples were stored at −80 °C until further steps.

### Profiling by TaqMan® Array Micro Fluidic Cards

We used the TaqMan^®^ MicroRNA Reverse Transcription Kit and related Megaplex™ RT Primers to synthesize single-stranded cDNA from the small RNA samples. For a full miRNA profile, we performed two separate reverse transcription reactions for each sample using pool A or pool B Megaplex^TM^ RT primers and 250 ng of RNA as template. The DNA polymerase from the TaqMan® Universal PCR Master Mix amplifies the target cDNA using sequence-specific primers and probes on the TaqMan® Low Density Array (TLDA-, Life Technologies, Carlsbad, CA, USA) by 7900HT Fast Real Time PCR System (Applied Biosystem, Foster City, CA, USA). We assayed two, TLDA, pre-loaded 384-well microfluidic cards with 754 spotted assays specific to human miRNAs, for each sample with respect to the Megaplex^TM^ RT product A and B. Each card contains four control assays; 3 selected candidate endogenous control assays (RNU6, RNU44 and RNU48, the first being in quadruplicate) and 1 negative control assay. We performed relative quantification (ΔΔCt) using the Gene Expression Suite Software (Life Technologies, Carlsbad, CA, USA) to process the miRNA expression data, using RNU6 as control endogenous assay, automatic baseline settings and a threshold of 0.2. miRNAs with Ct ˃35 were considered as undetermined. Gene Expression Suite Software included the student’s t-test for sample group comparisons and built Volcano Plot comparing the size of the fold change (biological significance) to the statistical significance (p-value). miRNAs identified as dysregulated were examined with two different miRNA databases aiming to identify validated and predicted target genes and related pathways (http://miRTarBase.mbc.nctu.edu.tw/ and http://mirgate.bioinfo.cnio.es/miRGate/).

### Gene Ontology enrichment and Reactome pathway analysis

Gene Ontology enrichment analysis was performed with the PANTHER (Protein ANalysis THrough Evolutionary Relationships) classification system (http://www.pantherdb.org), using the identified 278 miRNA target genes as queries for the statistical overrepresentation test and the most updated (at the time of analysis) Homo sapiens genes annotations as reference set; only the over-represented biological process terms with a p-value < 0.05 have been chosen to be reported in the graphs.

Reactome pathway analysis was performed with the “Analysis tool” available on https://reactome.org website and using the same genes as queries and Homo sapiens genes as reference set; selected overrepresented pathways, with p-value < 0.05, have been selected for further analysis.

### Validation by Taqman^®^ MicroRNA Assays

For the 15 candidate miRNAs we performed reverse transcription using the TaqMan^®^ MicroRNA Reverse Transcription Kit (Life Technologies, Foster City, CA) and specific 5x RT primers to synthesize single-stranded cDNA from 10 ng of total RNA samples. Each RT product was amplified using the TaqMan® Universal Master Mix II No AmpErase® UNG (Life Technologies, Carlsbad, CA, USA) and the appropriate 20x TaqMan® MicroRNA Assays (probe ID available upon request) to evaluate miRNA expression on the 7500 Real Time PCR System (Software 2.01, Applied Biosystems, Foster City, CA, USA). miRNA expression levels were normalized to the average levels of the endogenous small RNA control *U6 snRNA* and referred to the relevant control samples.

### mRNA expression by q- PCR

First-strand cDNA was synthesized from 1 μg of total RNA with random hexamer primers using the First-Strand cDNA Synthesis kit (GE Healthcare, Little Chalfont, UK).

The expression of markers of MN progenitors *OLIG2* (Hs00300164_s1) and *TUBB3* (Hs00801390_s1) was evaluated by quantitative real-time PCR analysis by means of the ΔΔCt method on a 7500 Real Time PCR System (Software 2.01, Applied Biosystems, Foster City, CA, USA).

We assayed the gene expression of *STX1A* (Hs00270282_m1), *SIRT1* (Hs01009006_m1), *TP53* (Hs01034249_m1), *SYT1* (Hs00194572_m1), *XIAP* (Hs00745222_s1), *MDM2* (Hs00540450_s1) and *ATF3* (Hs00231069_m1) by quantitative real-time PCR analysis by means of the ΔΔCt method. The expression levels of each gene were normalized to the average levels of the housekeeping gene *18S* (Hs99999901_s1) and referred to the relevant control samples.

### Statistical analysis

Statistical analyses were performed in the GraphPad Prism 5 software. All q-PCR counting data were expressed as mean the with SEM. The two-tailed, unpaired Student’s t test was utilized to compare two groups. The null hypothesis was rejected at a level of 0.05.

## Electronic supplementary material

Supplementary figures and tables
