# Supplementary material for: MicroRNA expression analysis identifies a subset of downregulated miRNAs in ALS motor neuron progenitors
Source: Sci Rep. 2018 Jul 4;8:10105. doi: 10.1038/s41598-018-28366-1 (PMC6031650; doi:10.1038/s41598-018-28366-1)
Supplement: Supplementary file 1 — Supplementary figures and tables [file 41598_2018_28366_MOESM1_ESM.doc]

**MicroRNA expression analysis identifies a subset of downregulated miRNAs in ALS motor neuron progenitors**

Mafalda Rizzuti^1^, Giuseppe Filosa^2^, Valentina Melzi^1^, Luca Calandriello^1^, Laura Dioni^3^, Valentina Bollati^3^, Nereo Bresolin^1^, Giacomo Pietro Comi^1^, Silvia Barabino^2^, Monica Nizzardo^1^, Stefania Corti^1*^

**Supplementary Figures**


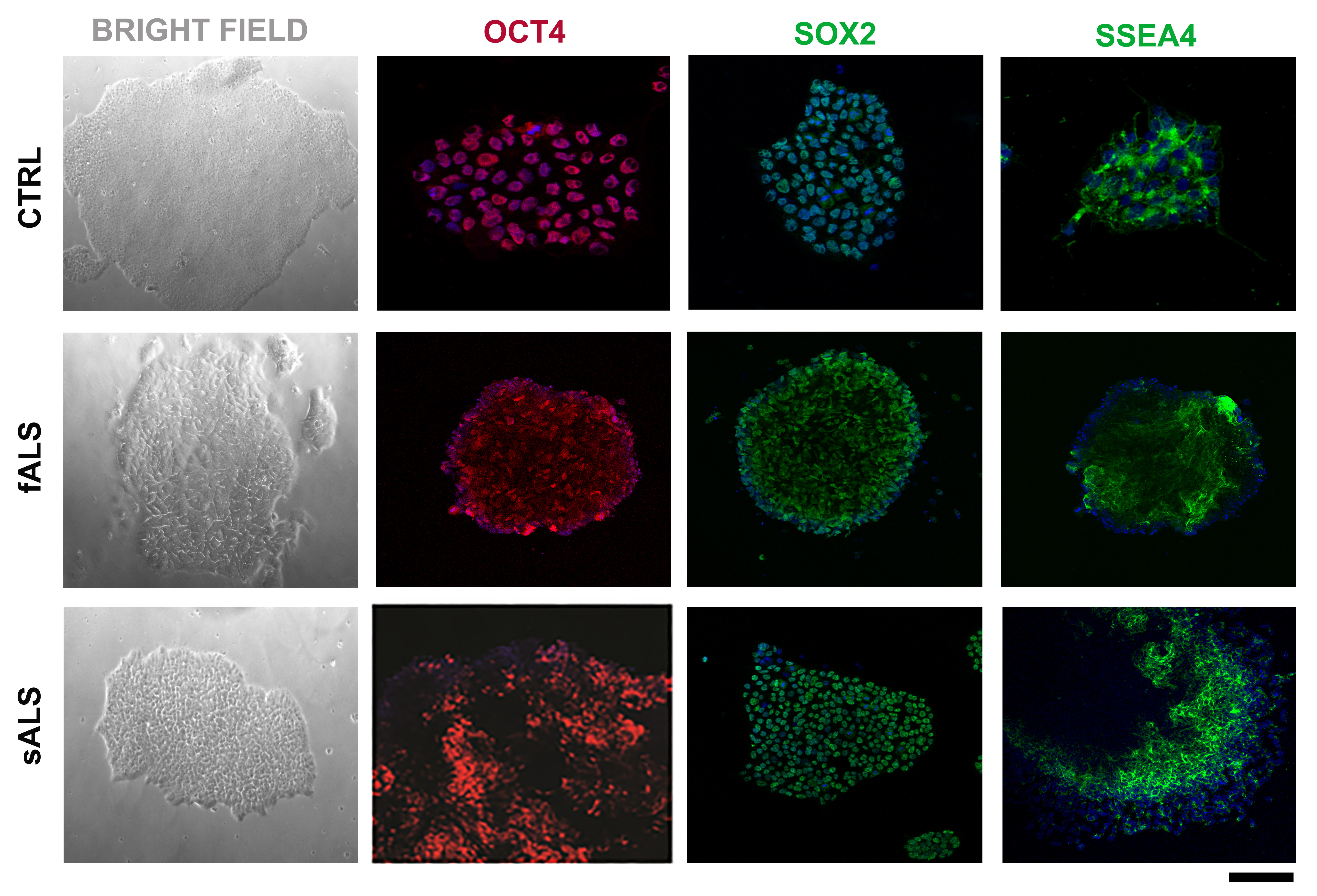


**Supplementary Figure S1: Immunocytochemistry (ICC) performed on iPSCs.**

Bright field and ICC for stem cells markers (OCT4, red; SOX2, green; SSEA4, green) on iPSCs generated from control (CTRL), fALS and sALS fibroblasts using a non-integrating reprogramming protocol. Nuclei are counterstained with DAPI (blue signal). Scale bars: bright field, ICC fALS and sALS 85 µm; ICC CTRL 65 µm


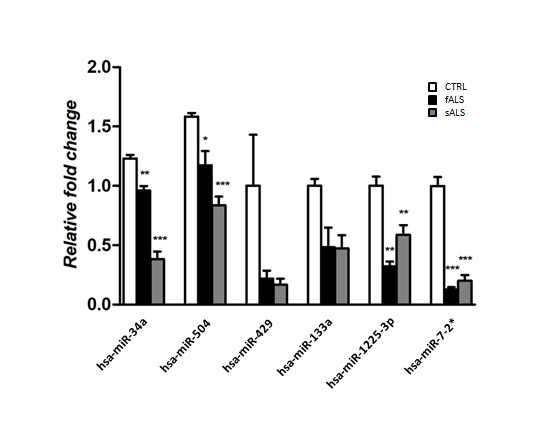


**Supplementary Figure S2: MiRNA expression profile in fALS and sALS patients versus healthy controls.**

Specific qPCR assays confirmed the downregulation of miR-34a, miR-504, miR-429, miR-133a, miR-1225-3p, and miR-7-2* in both fALS- and sALS-MN progenitors (****P* < 0.001, ***P* < 0.01 and **P* < 0.05, student t-test, values represent means + SEM) compared to the controls (CTRL).

**Supplementary Tables**


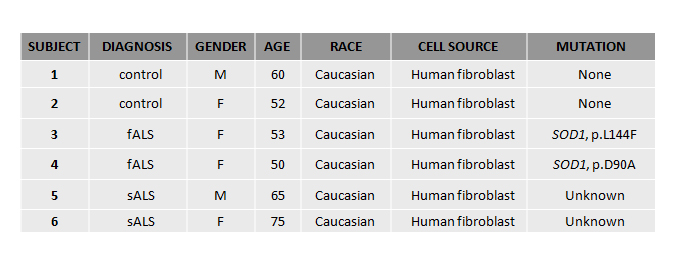


**Supplementary Table S1: Characteristics of human fibroblast-derived ALS induced pluripotent stem cell (iPSC) lines and controls.**

**
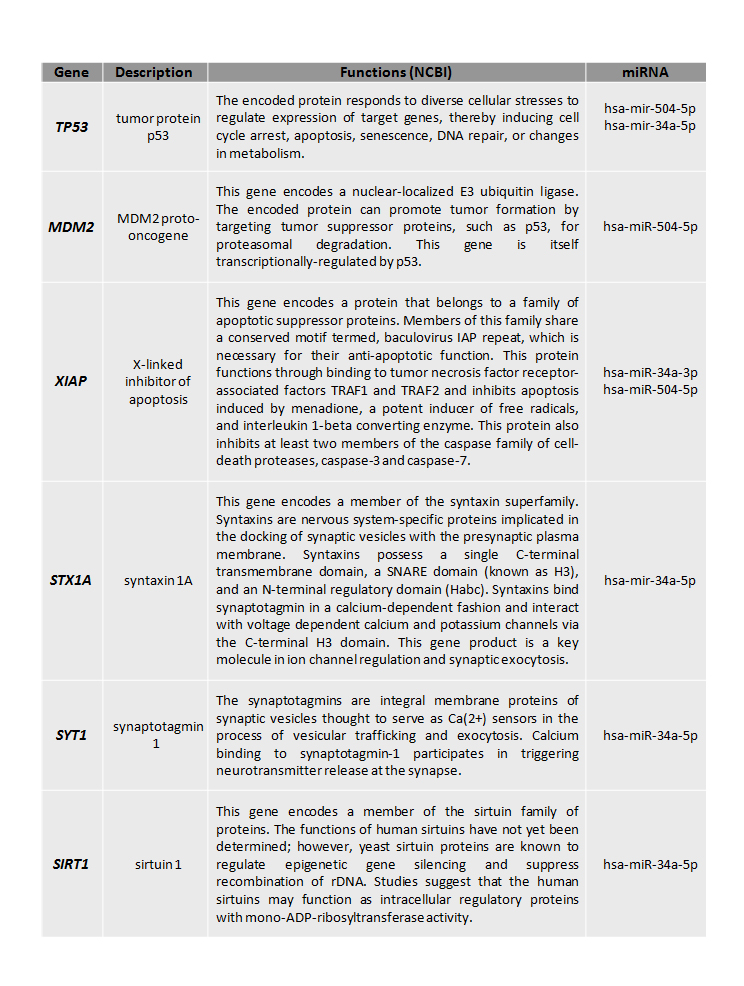
**

**Supplementary Table S2: Table of the most significant target genes and related pathways regulated by miR-34a and miR-504.**
